# Effect of Transgenic LD05 Maize Harboring the *m2cryAb-vip3A* Fusion Gene on Biodiversity in the Field

**DOI:** 10.3390/plants15213329

**Published:** 2026-10-28

**Authors:** Xinwei Hou, Wenlan Li, Guoqi Yao, Hongwei Sun, Fan Li, Runqing Yue

**Affiliations:** 1 Shandong Key Laboratory of Maize Biological Breeding, National Engineering Research Center of Wheat and Maize, Maize Research Institute, Shandong Academy of Agricultural Sciences, Jinan 250100, China; 15953852159@163.com (X.H.);; 2 Shandong Key Laboratory of Plant Virology, Institute of Plant Protection, Shandong Academy of Agricultural Sciences, Jinan 250100, China

**Keywords:** *Bacillus thuringiensis*, environmental risk assessment, lepidopteran insect resistance, non-target arthropods, weed community

## Abstract

The commercial application of genetically modified maize requires systematic assessment of its potential impacts on farmland biodiversity, which has become a core task in environmental safety evaluation. A two-year, single-site randomized complete block experiment with three replicate plots per treatment was conducted using LD05 carrying the *m2cryAb-vip3A* fusion gene. By direct observation and suction sampling, 54 arthropod species belonging to 12 orders and over 30 families were identified. LD05 exhibited significant resistance to target lepidopteran pests. For non-target arthropods, no statistically significant differences were observed between LD05 and Zheng58 in species richness, Shannon–Wiener diversity index, Simpson index, and Pielou index, and the population fluctuation trends of herbivorous pests and natural enemies were consistent between the two maize types, indicating no significant differences in non-target arthropod community structure. Under herbicide-free conditions, no differences in weed communities were found between LD05 and Zheng58. There was no significant difference in the occurrence of major diseases (*p* > 0.05). NMDS ordination revealed no obvious separation in the integrated community structure of insects, weeds, and diseases between LD05 and Zheng58 in 2023 (R^2^ = 0.280, *p* = 0.3) and 2024 (R^2^ = 0.119, *p* = 0.6). In summary, compared with Zheng58, no differences were detected for LD05 in the endpoints measured (arthropod diversity, weed communities, and disease incidence) under these conditions.

## 1. Introduction

Maize is one of the most important cereal crops worldwide, serving as a critical source of food, feed, and industrial raw materials [1,2]. However, it is chronically threatened by lepidopteran pests such as *Ostrinia furnacalis* (Guenée) and *Spodoptera frugiperda* (J. E. Smith), which cause direct yield losses and indirectly promote ear rot caused by fungal pathogens, reducing both yield and quality [3,4,5]. Annual yield losses due to insect pests range from 10% to 20% [6,7], and weed competition further exacerbates these losses [8,9]. Chemical pesticides have been the main strategy for controlling maize pests and weeds, but over-reliance has led to increased pest resistance, farmland pollution, and food safety risks [10,11,12]. Genetically modified (GM) insect-resistant and herbicide-tolerant maize offers a promising alternative by enabling precise pest control, reducing chemical pesticide use, and improving weed management [13].

GM insect-resistant maize has become an important strategy for managing lepidopteran pests globally [14]. Since the first transgenic maize event was approved for commercial cultivation in 1996 [15,16], more than 244 transformation events have been globally certified, of which 210 contain insect-resistance traits [2,3]. Major seed companies (Bayer and Corteva) developed single-gene transformation events such as MON810, TC1507, and MIR162 [3], which are conventionally pyramided to express multiple Bt proteins, thereby broadening the insecticidal spectrum and delaying resistance evolution. In 2025, China approved 96 GM maize varieties with stacked insect-resistance and herbicide-tolerance traits, marking a critical commercialization step (Ministry of Agriculture of the PRC (https://www.moa.gov.cn/) (accessed on 23 May 2026). Studies have shown that GM maize yields can be approximately 16.5% higher than non-transgenic varieties, and reduced insecticide use promotes biotic conservation [17,18,19,20]. However, the potential impacts on environmental safety still warrant attention [19,21,22]. Coupled with social concerns, and biosafety assessment requirements, the application of this crop in most regions of Asia and Africa faces numerous obstacles [23,24,25,26]. Therefore, systematic assessments of farmland biodiversity are needed.

Arthropods and weeds are key components of farmland ecosystems. Arthropods mediate essential ecosystem functions such as pollination, pest suppression, and organic matter decomposition [27,28,29], while weeds provide habitats for arthropods and maintain food-web structures [30,31]. Their communities require systematic evaluation in environmental risk assessments [31,32,33]. Previous studies generally found limited effects of insect-resistant maize on non-target arthropods [34,35,36,37,38,39]. LD05 is a novel transgenic maize line carrying the *m2cryAb-vip3A* fusion gene [40,41] and has been granted a biosafety certificate. While LD05 does not significantly affect soil rhizosphere microbial community [42], its impact on field biodiversity remains poorly understood. Therefore, this study systematically compared differences between LD05 and Zheng58 in 2023 and 2024 in arthropod community diversity and population dynamics, weed types and quantities, and major disease occurrence, and evaluated its field insect resistance and herbicide tolerance. This study aims to provide a scientific basis for the environmental safety evaluation and commercial application of LD05 in China.

## 2. Results

### 2.1. Variation in Arthropod Taxa and Abundance Between LD05 and Zheng58

**Figure 1:**
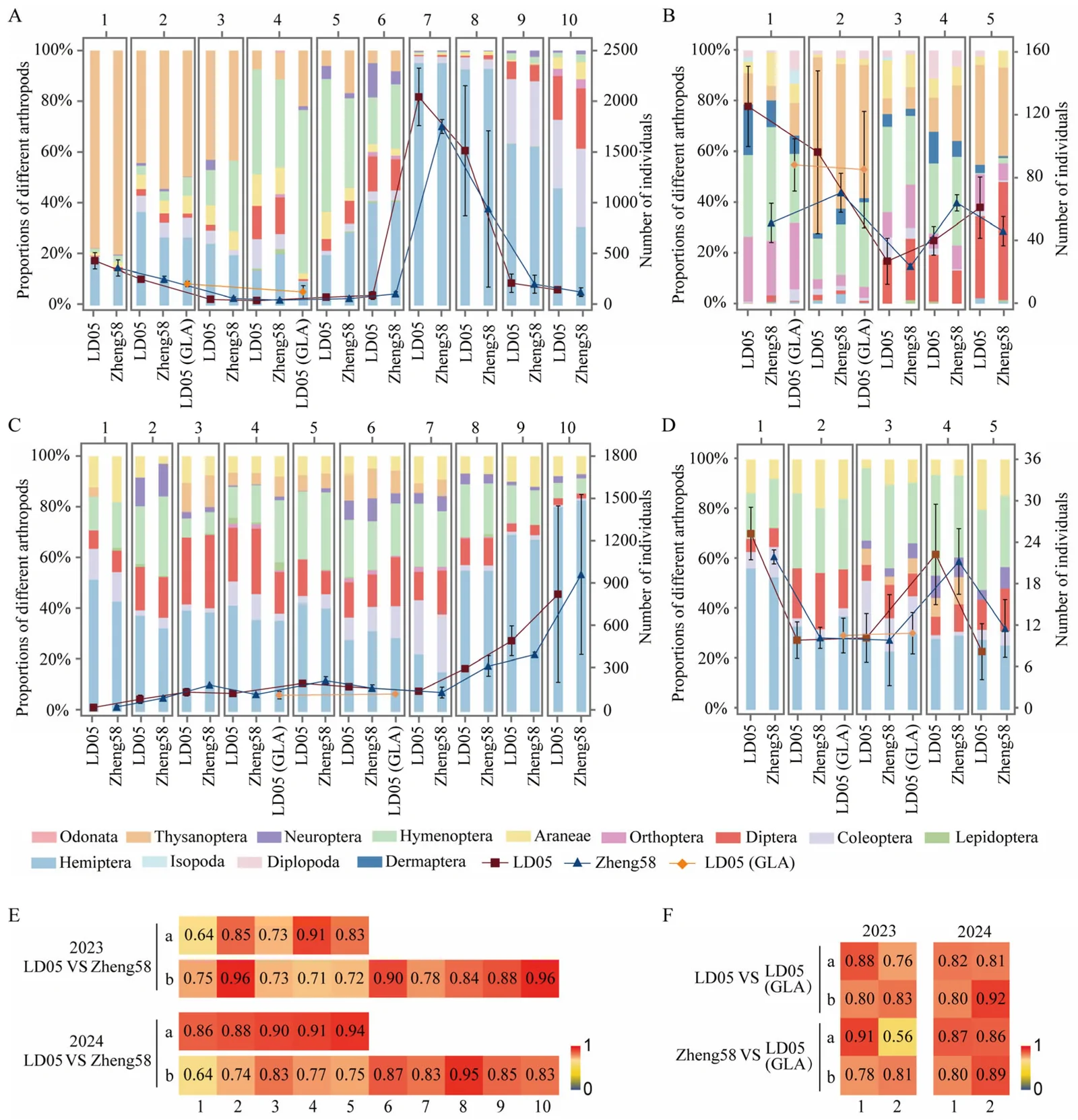
Arthropod taxa composition, and community similarity in LD05 and Zheng58 fields over 2023–2024. (**A**–**D**) The abundance and composition of arthropod taxa based on direct observation (**A**,**C**) and suction sampling (**B**,**D**) in 2023 (**A**,**B**) and 2024 (**C**,**D**). Percentages are calculated within each treatment-date-method group. Lines represent plot-level means (**E**) Community similarity indices (C′) between LD05 and Zheng58 fields across different sampling methods and years. (**F**) Community similarity indices (C′) between LD05(GA) and LD05 or Zheng58 fields. a and b represent suction sampling and direct observation, respectively. Numbers 1 to 10 indicate ten sampling events at seven-day intervals. Numbers 1 to 5 indicate five sampling events at ten-day intervals. For herbicide-treated plots (LD05(GA)), surveys were performed twice, at 2 and 4 weeks after application.

To assess the effects of transgenic maize LD05 on arthropod taxa and abundance, systematic field surveys of arthropod communities were conducted in 2023 and 2024 using direct observation (10 times per year) and suction sampling (5 times per year). Over the two years, direct observation recorded 27,598 (2023) and 16,180 (2024) arthropod individuals, while suction sampling captured 2320 (2023) and 520 (2024) individuals, respectively (Figure 1A–D) (Table S1). Although the species composition recorded each year was generally consistent, the relative abundance of individual species varied significantly between years. In terms of dominant taxa, in 2023, Hemiptera and Thysanoptera dominated both LD05 and Zheng58 fields, with maximum proportions reaching 95.22% and 95.19% (Hemiptera), and 77.48% and 80.00% (Thysanoptera) in the two maize types, respectively (Figure 1A). In 2024, the dominant taxa shifted to Hemiptera and Hymenoptera, with maximum proportions of 80.17% (LD05) and 82.82% (Zheng58) for Hemiptera, and 26.72% (LD05) and 30.26% (Zheng58) for Hymenoptera, respectively (Figure 1C). Suction sampling also revealed interannual differences. The number of arthropod taxa identified in 2024 (7 taxa) was notably lower than in 2023 (11 taxa), although Thysanoptera and Hymenoptera remained the dominant groups in both years, with maximum proportions of 69.69% (2023 Thysanoptera), 57.14% (2024 Thysanoptera), 20.68% (2023 Hymenoptera), and 8.68% (2024 Hymenoptera) (Figure 1B,D). Despite marked differences in arthropod abundance and taxa between years and sampling methods, at each sampling time point, there were no significant differences in arthropod taxa and abundance between LD05 and Zheng58, and the two maize fields exhibited similar arthropod population fluctuation trends throughout the survey period. The community similarity index (C′), which approaches 1.00 as similarity increases, was used to measure community resemblance. Over the two years, the community similarity indices between LD05 and Zheng58 were consistently relatively high (>0.6 for both sampling methods), indicating a high degree of community similarity between them. Meanwhile, the indices between LD05(GA) and LD05 or Zheng58 were all >0.55 (Figure 1E,F). These results indicate that arthropod communities in transgenic and non-transgenic maize fields were highly similar.

### 2.2. Variation in Arthropod Community Structure Between LD05 and Zheng58

**Figure 2:**
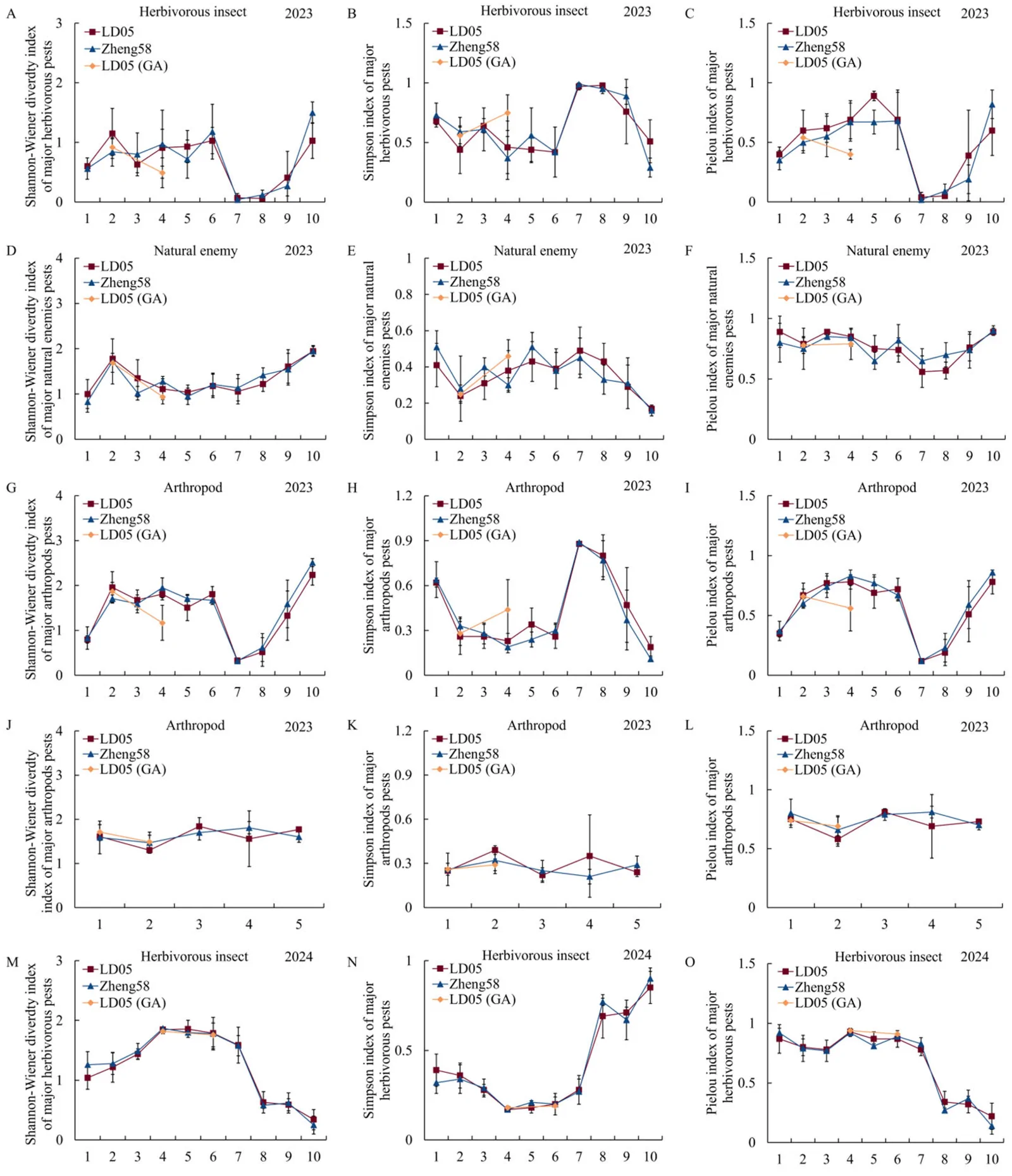

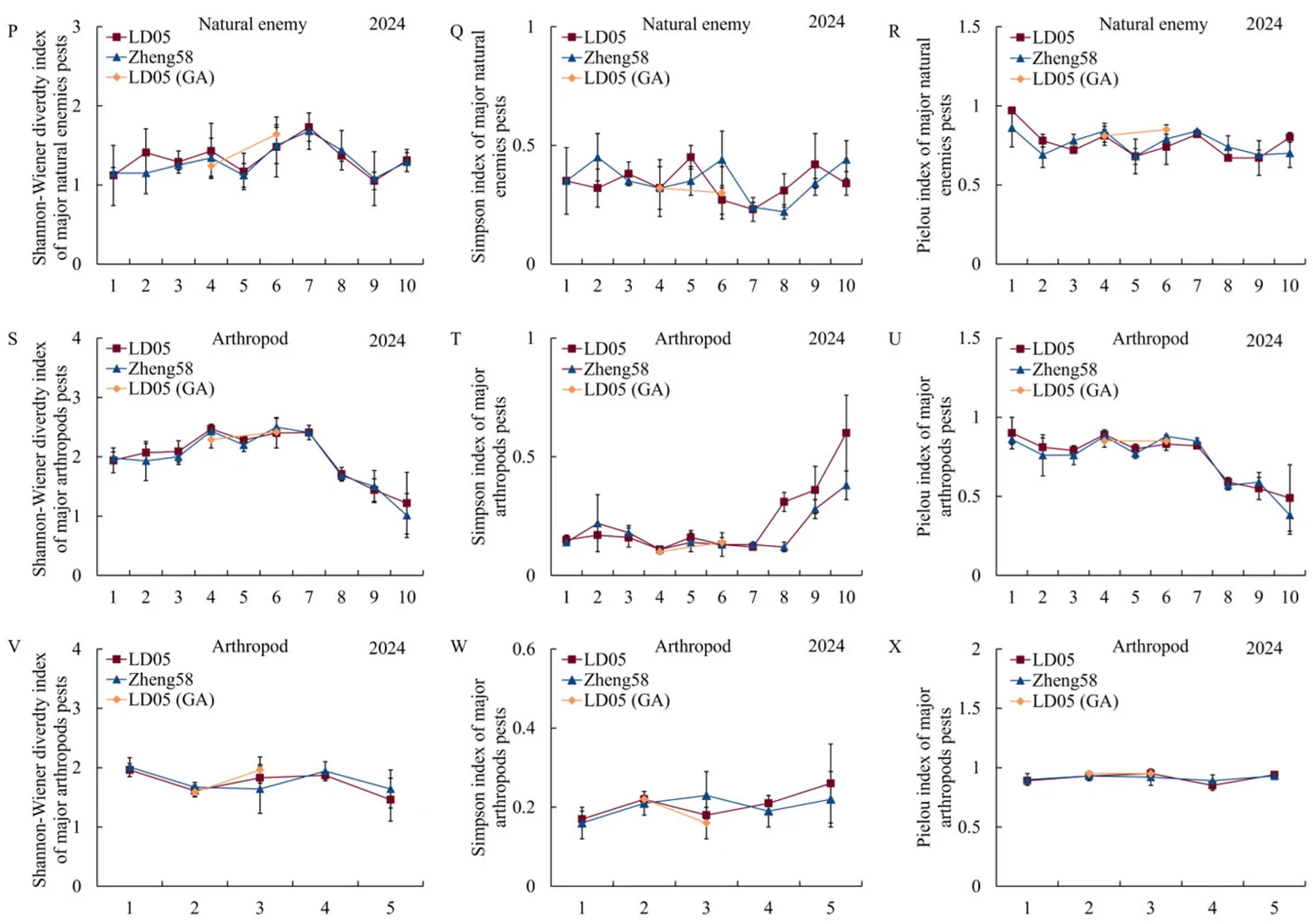
Ecological indices of arthropod communities in LD05 and Zheng58 fields. (**A**–**C**) Shannon–Wiener diversity indices (*H*), Pielou indices (*J*), and Simpson indices (*C*) of herbivorous insects based on direct observation in 2023; (**D**–**F**) *H*, *J*, and *C* of natural enemies based on direct observation in 2023; (**G**–**I**) *H*, *J*, and *C* of total arthropods based on direct observation in 2023; (**J**–**L**) *H*, *J*, and *C* of arthropods based on suction sampling in 2023; (**M**–**O**) *H*, *J*, and *C* of herbivorous insects based on direct observation in 2024; (**P**–**R**) *H*, *J*, and *C* of natural enemies based on direct observation in 2024; (**S**–**U**) *H*, *J*, and *C* of total arthropods based on direct observation in 2024; (**V**–**X**) *H*, *J*, and *C* of arthropods based on suction sampling in 2024. For herbicide-treated plots (LD05(GA)), surveys were performed twice, at 2 and 4 weeks after application. Other dates show only unsprayed LD05 and Zheng58. Error bars represent the standard error (SD) of the mean based on *n* = 3 independent replicate plots per treatment. Shannon–Wiener diversity index: the larger the value, the more complex the community composition. Pielou indices: the larger the value, the more evenly individuals are distributed among species in the community. Simpson dominance: larger values mean lower diversity.

To assess the effects of LD05 on arthropod community structure, direct observation and suction sampling were used for systematic monitoring of arthropod communities. The major herbivorous pests identified by direct observation were mainly from Hemiptera, Thysanoptera, and Diptera, while the dominant predatory natural enemies were primarily from Neuroptera, Hemiptera, and Coleoptera. the Shannon–Wiener diversity index (*H*), the Pielou index (*J*), the Simpson index (*C*) of herbivorous insects, natural enemies, and the total arthropod community were calculated and compared. The results showed that none of the three ecological indices for the three community groups differed significantly between LD05 and Zheng58 (Figure 2A–I,M–U). The arthropods collected by suction sampling mainly belonged to Hymenoptera, Araneae, Orthoptera, Coleoptera, and Dermaptera. Similarly, no significant differences were detected in the *H*, *J*, and *C* of the suction-sampled arthropods community between LD05 and Zheng58 (Figure 2J–L,V–X). After application of the target herbicide glufosinate, the three ecological indices of arthropod communities in LD05 plots remained comparable to those in unsprayed LD05 and Zheng58, with no significant differences observed (Figure 2A–X). Collectively, under the tested conditions, no statistically significant differences were detected in the diversity of field arthropod communities between transgenic maize LD05 and the control Zheng58, nor following the application of glufosinate.

### 2.3. Variation in Arthropod Community Dynamics Between LD05 and Zheng58

**Figure 3:**
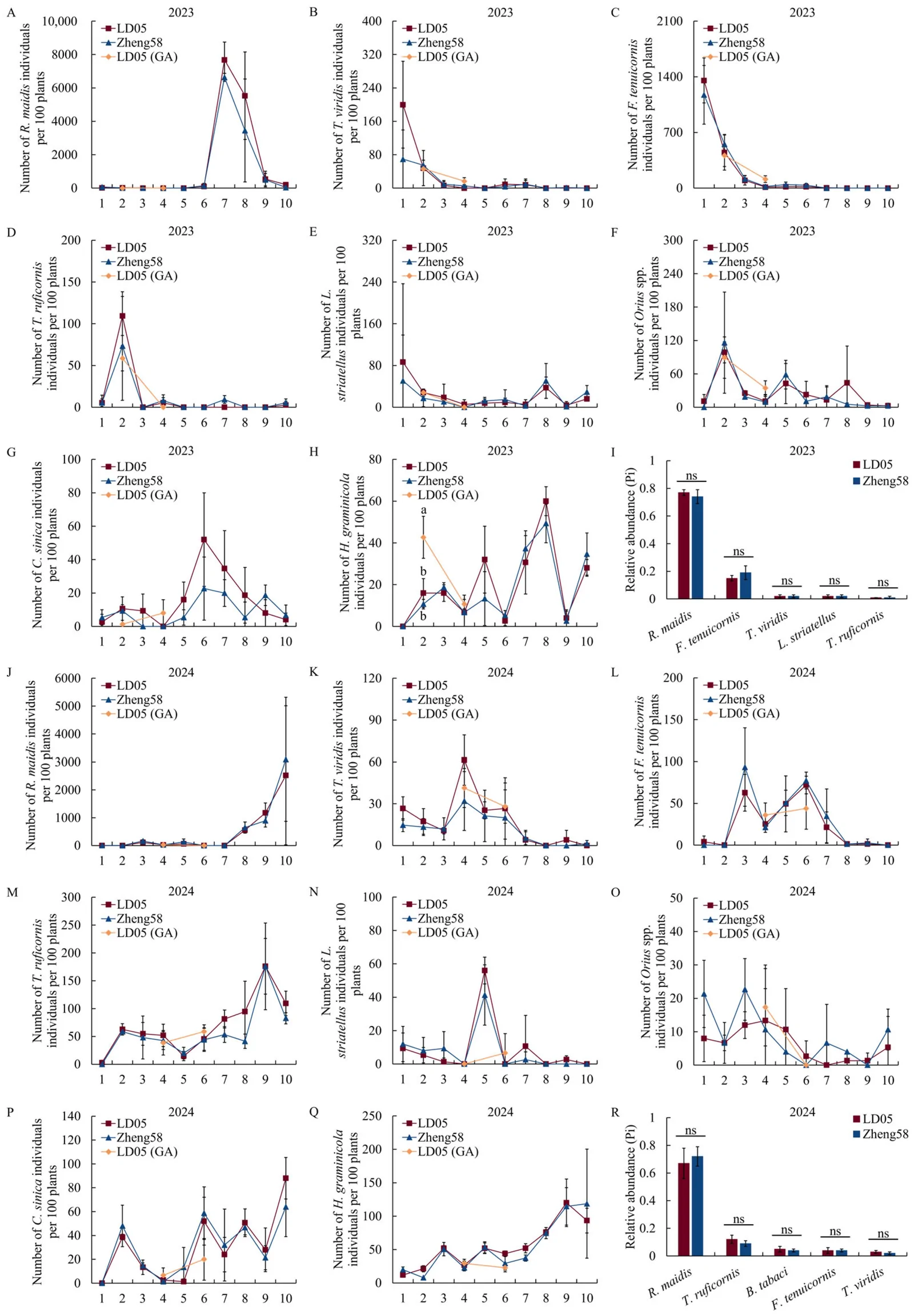
Population dynamics of major herbivorous pests and natural enemies in LD05 and Zheng58 fields during 2023 and 2024. (**A**–**I**) Data from 2023: (**A**–**H**) temporal abundance of *R. maidis*, *T. viridis*, *F. tenuicornis*, *T. ruficornis*, *L. striatellus*, *Orius* spp., *C. sinica*, and *H. graminicola* over 10 sampling time points; (**I**) relative abundance of dominant herbivorous insects. (**J**–**R**) Data from 2024: (**J**–**Q**) temporal abundance of *R. maidis*, *T. viridis*, *F. tenuicornis*, *T. ruficornis*, *L. striatellus*, *Orius* spp., *C. sinica*, and *H. graminicola* over 10 sampling time points; (**R**) relative abundance of dominant herbivorous insects. For herbicide-treated plots (LD05(GA)), surveys were performed twice, at 2 and 4 weeks after application. Error bars represent the standard deviation (SD) of the mean based on *n* = 3 independent replicate plots per treatment. Abundance per 100 plants = (individuals observed per plot/50) × 100. At the same sampling period, values with different lowercase letters indicate significant differences among treatments (*p* < 0.05). ns indicates no significant difference (*p* ≥ 0.05).

To investigate the effects of LD05 on arthropod population dynamics, the numbers of major herbivorous pests and natural enemy groups in each plot were systematically counted using direct observation in 2023 and 2024. The major herbivorous pests included *Rhopalosiphum maidis* (*R. maidis*) (Fitch) (Figure 3A,J), *Tettigella viridis* (*T. viridis*) (Linnaeus) (Figure 3B,K), *Frankliniella tenuicornis* (*F. tenuicornis*) (Uzel) (Figure 3C,L), *Trigonotylus ruficornis* (*T. ruficornis*) (Geoffroy) (Figure 3D,M), and *Laodelphax striatellus* (*L. striatellus*) (Fallén) (Figure 3E,N). The main natural enemy groups comprised *Orius spp.* (*O. spp.*) (Wollf) (Figure 3F,O), *Chrysoperla sinica* (*C. sinica*) (Tjeder) (Figure 3G,P), and *Hylyphantes graminicola* (*H. graminicola*) (Sundevall) (Figure 3H,Q). No significant difference was detected in the population fluctuation trends of herbivorous pests and natural enemies between LD05 and Zheng58. Application of the target herbicide glufosinate did not significantly affect the population dynamics of these herbivorous pests and predatory arthropods. The only exception was *H. graminicola* during the second survey, which was significantly higher in LD05(GA) (42.67 ± 10.06) than in LD05 (16.00 ± 6.93) and Zheng58 (10.67 ± 2.31). However, this isolated difference is likely attributable to sampling error and the aggregated distribution of insects under low population densities, rather than a true treatment effect. Across the two years, the dominant pest insects in 2023 were *R. maidis*, *F. tenuicornis*, *T. viridis*, *L. striatellus*, and *T. ruficornis* (Figure 3I); in 2024, they were *R. maidis*, *T. ruficornis*, *Bemisia tabaci* (*B. tabaci*) (Gennadius), *F. tenuicornis*, and *T. viridis* (Figure 3R). Further analysis of the relative abundance of dominant pest insects revealed no significant difference between LD05 and Zheng58.

### 2.4. Insect Resistance and Field Disease Occurrence of LD05

**Figure 4:**
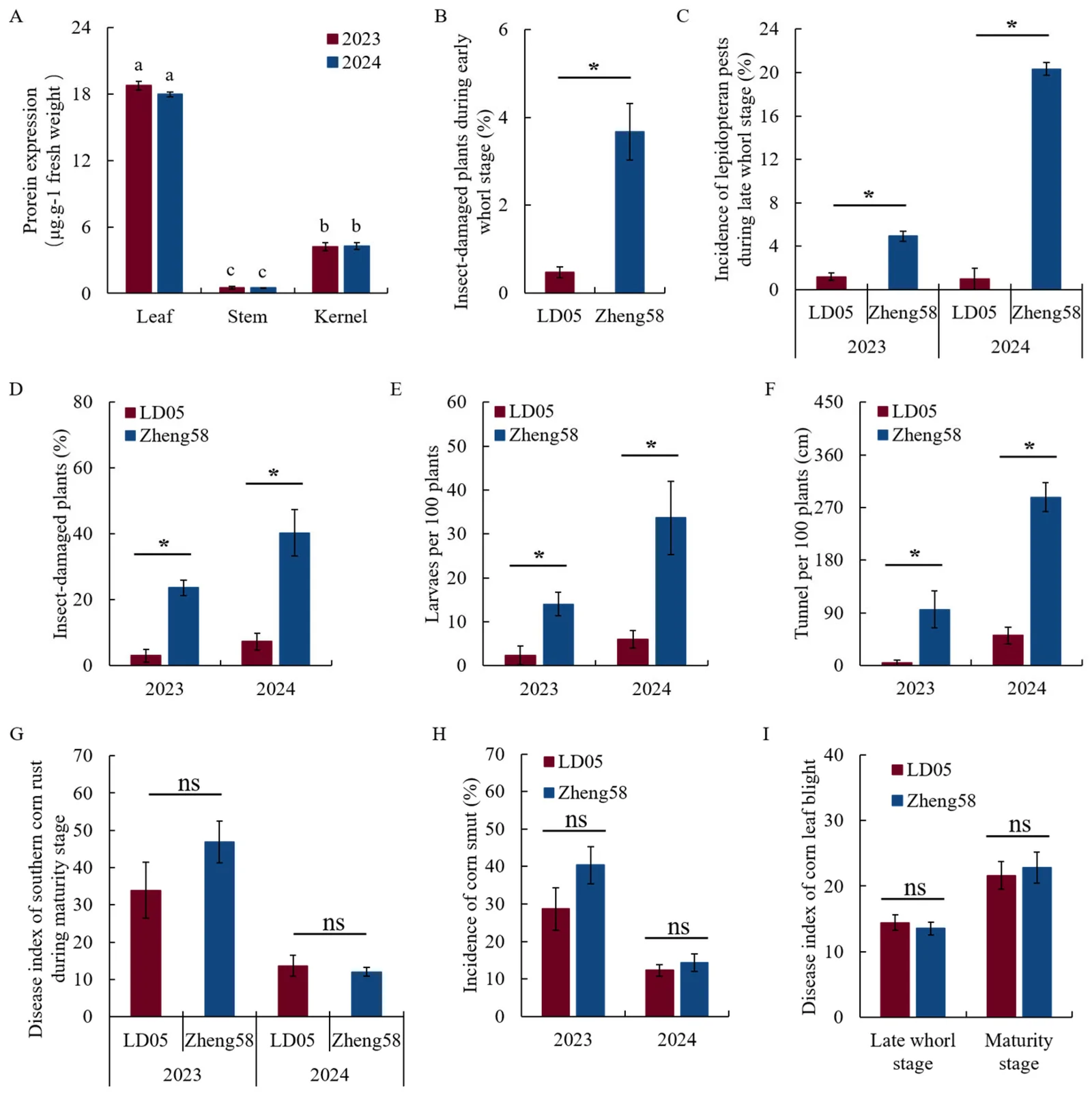
Resistance of transgenic maize LD05 to target lepidopteran pests and disease occurrence in the field. (**A**) Accumulation levels of M2CryAb-VIP3A protein in different tissues in 2023 and 2024. (**B**) Damaged plant rate at the early whorl stage in 2024. (**C**) Damaged plant rate at the late whorl stage in 2023 and 2024. (**D**) Damaged plant rate at maturity in 2023 and 2024. (**E**) Number of larvae per 100 plants in 2023 and 2024. (**F**) Stalk tunnel length (cm) in 2023 and 2024. (**G**) Disease indices of southern rust in 2023 and 2024. (**H**) Incidence of corn smut in 2023 and 2024. (**I**) Disease indices of leaf spot at the late whorl stage and maturity in 2024. Error bars represent the standard deviation (SD) of the mean based on *n* = 3 independent replicate plots per treatment. Asterisks above the error bars indicate significant differences (*p* < 0.05). Different lowercase letters indicate significant differences (*p* < 0.05), and “ns” indicates no significant difference.

To verify the resistance of transgenic maize LD05 to target lepidopteran pests, the accumulation levels of the target protein M2CryAb-VIP3A were detected in leaves at the whorl stage (V6–V7 stage) and in stalks and kernels at maturity in both 2023 and 2024. The M2CryAb-VIP3A protein was measured in leaves at the whorl stage and in stalks and kernels at maturity because these tissues and stages correspond to those used for insect resistance assessment, thereby providing mutual corroboration of the insect-resistant performance of LD05. The results showed that the protein accumulated most abundantly in whorl-stage leaves, followed by kernels, and least in stalks, while no protein was detected in Zheng58, with concentrations in leaves, stems, and kernels of 18.78 ± 0.40, 0.52 ± 0.10, and 4.22 ± 0.39 μg/g fresh weight in 2023 and 17.99 ± 0.23, 0.49 ± 0.03, and 4.28 ± 0.30 μg/g fresh weight in 2024, respectively. (Figure 4A) (Table S2). In 2023, no lepidopteran pest damage was observed at the early whorl stage; however, at the late whorl stage, the damaged plant rate of LD05 (1.00 ± 1.00%) was significantly lower than that of Zheng58 (4.93 ± 0.46%) (Figure 4C) (Table S4). In 2024, lepidopteran pest damage was detected at both the early and late whorl stages, and the damaged plant rates of LD05 (0.47 ± 0.12% and 1.20 ± 0.35%) were significantly lower than those of Zheng58 (3.67 ± 0.64% and 20.33 ± 0.58%) (Figure 4B,C) (Table S4). At maturity, the damaged plant rates of LD05 in 2023 and 2024 (3.00 ± 2.00% and 7.33 ± 2.52%) were significantly lower than those of Zheng58 (23.67 ± 2.31% and 40.33 ± 7.02%) (Figure 4D) (Table S4). In addition, the average number of larvae per 100 plants in LD05 (2.33 ± 2.08 and 6.00 ± 2.00) was significantly lower than that in Zheng58 (14.00 ± 2.65 and 33.67 ± 8.39) (Figure 4E) (Table S5), and the stalk tunnel length (5.00 ± 4.58 cm and 51.33 ± 14.22 cm) was also significantly shorter than that of Zheng58 (95.67 ± 31.75 cm and 287.67 ± 25.03 cm) (Figure 4F) (Table S6). These results indicate that, under field conditions, LD05 significantly reduced damage of the naturally occurring target lepidopteran pests compared to the control.

Regarding disease occurrence, no diseases were recorded at the late whorl stage in 2023; at maturity, southern rust and corn smut were the predominant diseases. No significant differences were detected between LD05 and Zheng58 in the disease severity index of southern rust (2023: 33.93 ± 7.45% vs. 46.89 ± 5.63%; 2024: 13.7 ± 2.72% vs. 12.07 ± 1.14%) (Table S7) and leaf spot (21.63 ± 2.14% vs. 22.81 ± 2.4%) (Table S9) or in the incidence of corn smut (2023: 28.67 ± 5.69% vs. 40.33 ± 5.03%; 2024: 12.33 ± 1.53% vs. 14.33 ± 2.31%) (Figure 4G–I) (Table S8). In 2024, at the late whorl stage, the disease index of leaf spot in LD05 did not differ significantly from that of Zheng58 (14.44 ± 1.15% vs. 13.56 ± 0.97%) (Figure 4I) (Table S9). In summary, no statistically significant differences were detected in the occurrence of the major field diseases surveyed between transgenic maize LD05 and the control under the tested conditions.

### 2.5. Effects of LD05 Cultivation and Glufosinate Application on Field Weeds

**Figure 5:**
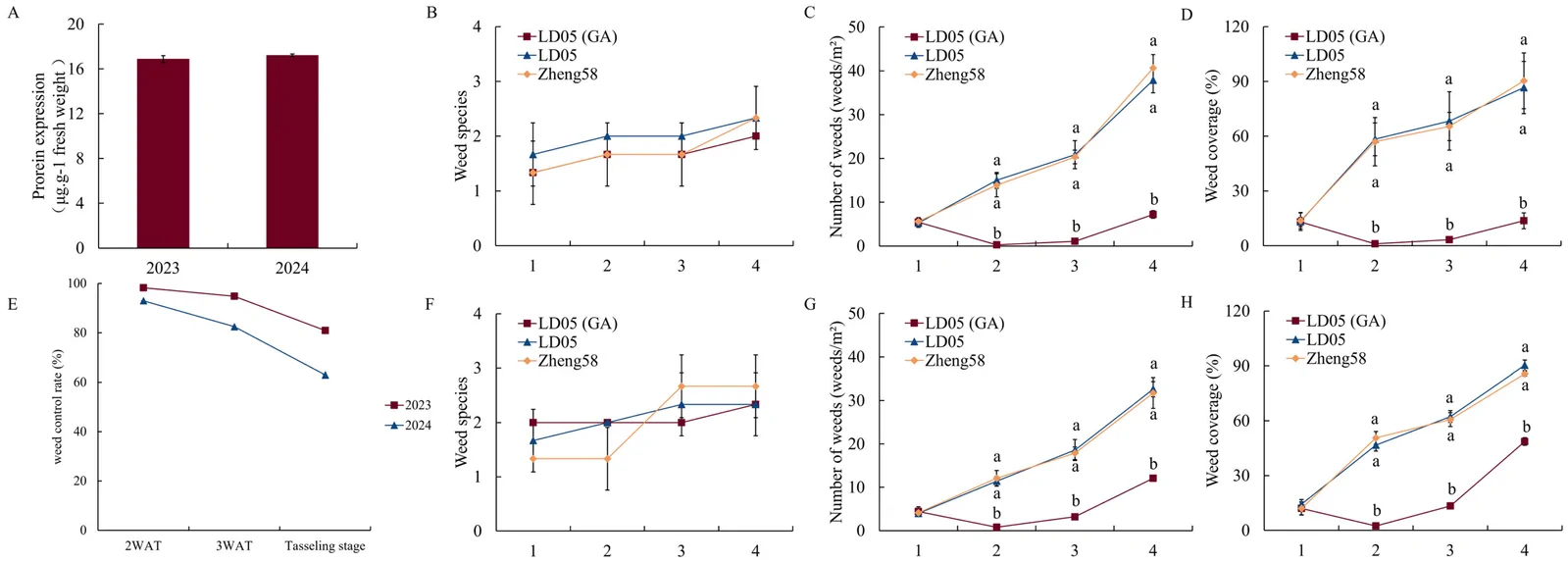
Effects of LD05 cultivation and glufosinate application on field weed communities. (**A**) PAT protein accumulation in leaves at the whorl stage, detected by ELISA. (**B**–**D**) Temporal dynamics of weed species number (**B**), weed abundance (**C**), and weed coverage (**D**) in three treatments (LD05, LD05 (GA), and Zheng58) during the 2023 growing season. (**F**–**H**) Temporal dynamics of weed species number (**F**), weed abundance (**G**), and weed coverage (**H**) in the three treatments during the 2024 growing season. (**E**) Weed control efficacy of glufosinate applied at the whorl stage (V6–V7) in 2023 and 2024, assessed at three time points after application. WAT, weeks after treatment; 2 WAT, 2 weeks after glufosinate application; 3 WAT, 3 weeks after glufosinate application; tasseling stage (VT). Error bars represent the standard deviation (SD) of the mean based on *n* = 3 independent replicate plots per treatment. During the same survey period, values followed by the same lowercase letter indicate no significant difference among different maize varieties according to Duncan’s test (*p* ≥ 0.05), whereas values with different lowercase letters indicate significant differences (*p* < 0.05).

To investigate the effects of transgenic maize LD05 cultivation and glufosinate application on field weeds, the accumulation of PAT protein in leaves at the whorl stage was examined. PAT protein was expressed in LD05 leaves at the whorl stage in both 2023 and 2024, with concentrations of 16.87 ± 0.32 and 17.21 ± 0.12 μg/g fresh weight, respectively (Figure 5A) (Table S3). Before glufosinate application, no significant differences were observed in weed species, abundance, or coverage among the LD05 plots designated for glufosinate application (LD05 (GA)), the herbicide-free LD05 plots, and Zheng58 plots (Figure 5B–D,F–H). After glufosinate application at the whorl stage (V6–V7), the control efficacy in 2023 was 98.25 ± 0.01%, 94.77 ± 0.02%, and 80.88 ± 0.03% over three surveys, while in 2024 it was 92.90 ± 0.02%, 82.44 ± 0.05%, and 62.88 ± 0.03%, respectively (Figure 5E) (Table S10). Following herbicide application, weed species remained unchanged (Table S11), but weed abundance and coverage were significantly reduced in LD05(GA) plots compared to LD05 and Zheng58 fields. (Figure 5B–D,F–H). In 2023, at the three survey dates, the weed number (weeds/m^2^) in LD05(GA) plots was 0.27 ± 0.12, 1.07 ± 0.31, and 7.20 ± 0.87, which was significantly lower than that in LD05 (15.00 ± 1.80, 20.87 ± 3.20, and 37.87 ± 2.86) and Zheng58 (13.87 ± 2.64, 20.33 ± 1.55, and 40.66 ± 3.06). Similarly, weed coverage in LD05(GA) plots was merely 1.00 ± 0.00%, 3.33 ± 0.58%, and 13.67 ± 1.53%, which was significantly lower than those in LD05 (58.33 ± 4.51%, 68.33 ± 4.04%, and 86.67 ± 4.04%) and Zheng58 (57.00 ± 3.46%, 65.33 ± 1.15%, and 90.33 ± 4.16%) (Tables S12 and S13). In 2024, weed number (weeds/m^2^) in LD05(GA) plots (0.80 ± 0.20, 3.20 ± 0.53, and 12.06 ± 0.64) remained significantly lower than that of LD05 (11.40 ± 1.11, 18.60 ± 2.43, and 32.60 ± 1.74) and Zheng58 (12.07 ± 1.81, 17.87 ± 1.45, and 31.73 ± 3.53). The weed coverage in LD05(GA) plots (2.33 ± 0.58%, 13.33 ± 1.53%, and 48.67 ± 2.08%) was also significantly reduced compared to LD05 (46.67 ± 3.21%, 62.33 ± 3.21%, and 90.33 ± 2.89%) and Zheng58 (50.67 ± 3.51%, 60.67 ± 3.79%, and 85.67 ± 1.15%) (Tables S12 and S13). At all survey times, no significant differences were detected in weed species, abundance, or coverage between unsprayed LD05 and Zheng58 fields, indicating that cultivation of transgenic maize LD05 itself did not affect weed species, abundance, and coverage (Figure 5B–D,F–H). In summary, glufosinate application at the whorl stage effectively suppressed field weeds. However, in the absence of herbicide application, no statistically significant differences were detected in weed species, abundance, or coverage between LD05 and Zheng58 fields under the tested conditions.

### 2.6. Comparison of Community Traits Between LD05 and Zheng58

**Figure 6:**
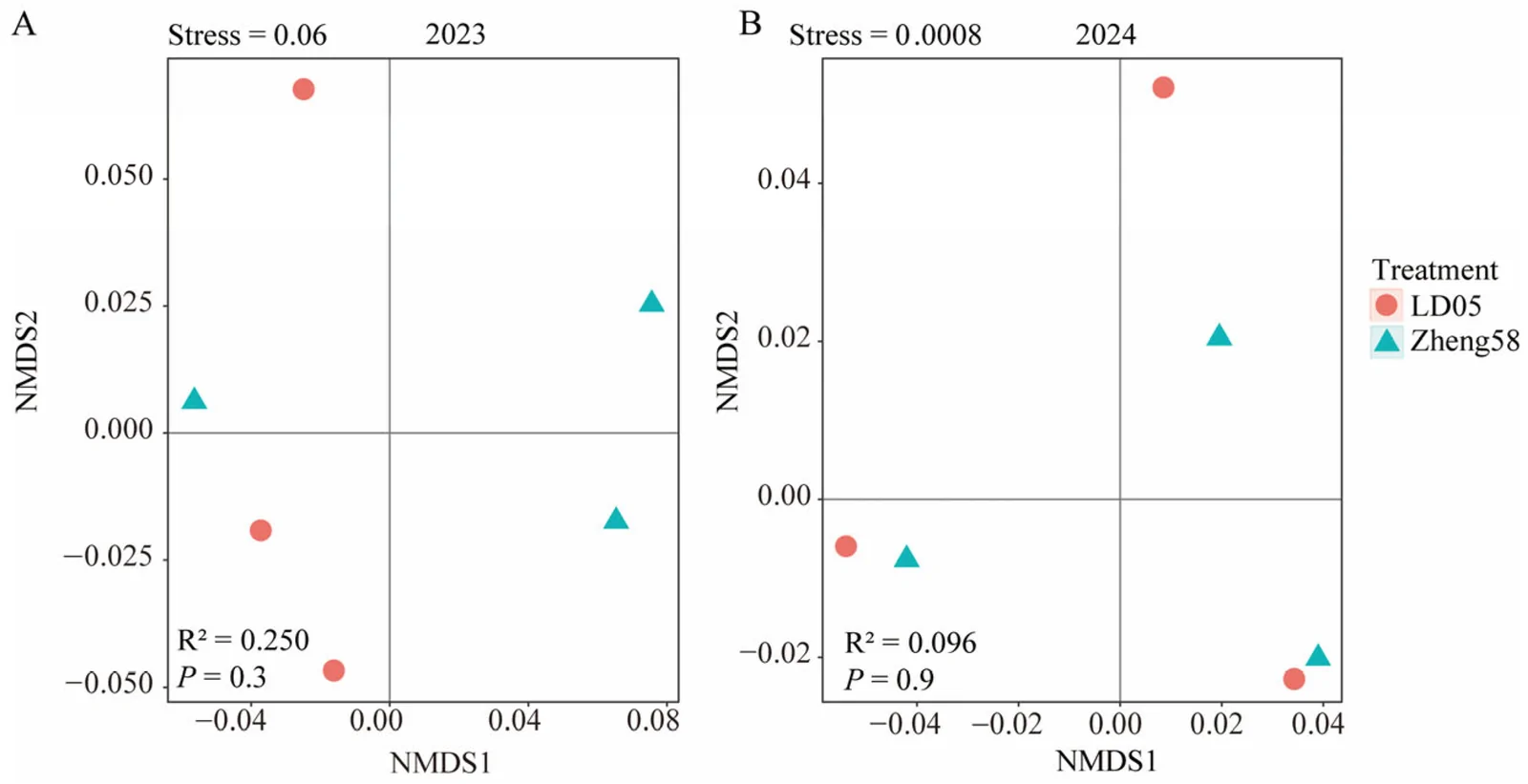
Non-metric multidimensional scaling (NMDS) analysis of integrated community structure (arthropods, weeds, and diseases) in LD05 and Zheng58 fields. (**A**) NMDS ordination plot for the 2023 dataset; (**B**) NMDS ordination plot for the 2024 dataset. Green triangles and orange circles represent Zheng58 and LD05. Community distances were calculated using the Bray–Curtis dissimilarity index. The stress values (<0.2) indicate acceptable ordination configurations.

To comprehensively evaluate the effects of transgenic maize LD05 on field community structure, arthropod community parameters, weed community characteristics, and disease incidence were integrated as comprehensive community traits of the maize field ecosystem. The data matrix consisted of 6 independent plots (3 plots per treatment per year × 2 treatments). Percentage variables were transformed using the arcsine square-root transformation, and count variables were transformed using the square root transformation. To combine measurements collected at different dates into a single row per plot, data from all sampling dates were averaged within each plot. Non-metric multidimensional scaling (NMDS) was employed to compare the community structures between transgenic maize LD05 and Zheng58, with community distances estimated based on the Bray–Curtis dissimilarity index. PERMANOVA analysis revealed no significant differences in community structure between LD05 and Zheng58 in 2023 (pseudo-F = 1.335, df = 1, 4, R^2^ = 0.250, *p* = 0.3) (Figure 6A) or 2024 (pseudo-F = 0.424, df = 1, 4, R^2^ = 0.096, *p* = 0.9) (Figure 6B). The permutation test for multivariate dispersion revealed no significant differences between LD05 and Zheng58 in either 2023 (F = 0.137, df = 1, 4, *p* = 0.501) or 2024 (F = 0.618, df = 1, 4, *p* = 0.401). The absence of a significant dispersion effect indicates that the PERMANOVA results were not confounded by unequal dispersion (Code S1). These results indicate that no statistically significant difference was detected in the integrated community characteristics between LD05 and Zheng58 under the tested conditions.

## 3. Discussion

Since its commercialization in 1996, transgenic maize traits have evolved from single insect resistance or herbicide tolerance to stacked traits combining both insect resistance and herbicide tolerance [43]. Current mainstream varieties typically integrate insect-resistance and herbicide-tolerance genes through gene stacking technologies, but mainly rely on hybridization-based pyramiding [44,45]. In contrast, LD05 in this study carries the insect-resistant fusion gene *m2cryAb-vip3A* with independent intellectual property rights, achieving the innovation of a single insect-resistance gene with dual protein activities (*Cry1Ab* and *Vip3Aa*), thereby broadening the insecticidal spectrum and enhancing insect resistance [40]. Results from most experimental risk assessments have shown that the occurrence of target lepidopteran pests and the increase in their pressure levels have significant impacts on the ecological environment [46,47,48]. In this study, due to the expression of the M2CryAb-VIP3A protein at different developmental stages of LD05, target lepidopteran pests were significantly controlled in multi-year field experiments, with insect pressure maintained at extremely low levels. The protein content showed similar trends across different tissues, in agreement with previously reported results [40]. This fully demonstrates the excellent insect resistance of LD05 against lepidopteran pests in field trials.

Although transgenic insect-resistant and herbicide-tolerant maize brings significant economic benefits while effectively controlling target pests, its potential ecological risks—particularly the long-term effects on non-target arthropods—have gradually become a core issue in environmental safety assessment, and this concern continues to deepen with the extension of planting years and expansion of cultivation area [13,49]. Therefore, field biodiversity assessment is a key component of environmental safety evaluation for transgenic crops. In this study, direct observation and suction sampling methods were used to systematically identify arthropod communities. In the maize fields of Zhangqiu District, Jinan City, Shandong Province, a total of 54 arthropod species were identified, belonging to 12 orders and over 30 families. There were considerable differences in arthropod species and abundance between years, which is consistent with previous field surveys, indicating that arthropod species richness fluctuates significantly across years and months, likely attributable to the synergistic regulation of multiple factors in the agroecosystem, including food resource uniformity, temperature and humidity variations, rainfall distribution, and natural enemy abundance [38,50,51]. However, there were few differences in arthropod species and abundance between LD05 and Zheng58, which had high community similarity indices. This indicates that, compared with the background variation driven by climatic factors, the impact of transgenic maize cultivation on field arthropod communities was relatively minor. Current research on the effects of transgenic crops on field arthropods has mainly focused on two major groups: herbivorous pests and natural enemies. Herbivores generally exhibit higher population density and species richness than natural enemy groups in arthropod communities. No significant differences were observed between LD05 and Zheng58 in the composition and structure of non-target arthropod communities, and key ecological indices (*H*, *J* and *C*) did not differ significantly between the two systems. Furthermore, there were no differences in the relative abundance of herbivorous pests and natural enemies between LD05 and Zheng58 fields, and the population fluctuation trends of various insect groups remained largely consistent. The relative abundance of dominant pest insects was not affected by LD05 cultivation. This conclusion is consistent with earlier evidence obtained from other Bt crops such as DBN9936 and HGK60 [37,49].

The cultivation of transgenic herbicide-tolerant maize, in combination with glufosinate application, can effectively control field weeds. Particularly in areas with severe weed infestations and difficult weed management, planting herbicide-tolerant maize represents an important strategy for achieving efficient weed control. The transgenic insect-resistant and herbicide-tolerant maize LD05 used in this study exhibited glufosinate tolerance under field conditions, with PAT protein expression detected in the leaves at the whorl stage in both 2023 and 2024. Following glufosinate application, weeds were effectively controlled within a short period. Under herbicide-free conditions, no significant differences were observed in weed species composition, abundance, or coverage between LD05 and Zheng58. In addition, LD05 showed no significant differences from Zheng58 in terms of disease species, timing of occurrence, or severity.

As a major maize-producing region in China, the Huang-Huai-Hai area has long faced the dual pressures of lepidopteran pest outbreaks and high-intensity chemical pesticide inputs. The Zhangqiu pilot base, where this experiment was conducted, represents the typical farmland landscape of this region in terms of soil type, climatic conditions, and cropping system. By achieving effective control of target pests without the need for additional chemical insecticide sprays, LD05 could potentially reduce the potential harm of broad-spectrum insecticides to non-target arthropod communities. However, this study has several limitations. First, the experiment was conducted only at a single site in Zhangqiu, Shandong Province. Although this site is regionally representative, extrapolation of the conclusions still requires multi-year and multi-site validation across more ecological regions. Second, whether the long-term cultivation of LD05 will induce resistance evolution in target pests or cause secondary pest succession remains to be evaluated through long-term population dynamics monitoring.

In summary, under the tested conditions, transgenic insect-resistant and herbicide-tolerant maize LD05 demonstrated effective control against target pests, and no statistically significant differences were detected in non-target arthropod diversity, weed communities, or disease incidence compared to Zheng58. LD05 also exhibited glufosinate tolerance, effectively suppressing weed growth in the field. However, the limited sample size (three replicate plots per treatment) and single-site, two-season design restrict broad extrapolation. Therefore, this study provides baseline ecological data under the tested conditions, which may serve as a reference for the safety and commercial application of LD05.

## 4. Materials and Methods

### 4.1. Plant Materials and Field Experimental Design

Transgenic maize LD05 and the non-transgenic maize Zheng58 (Control) were provided by the Maize Research Institute of Shandong Academy of Agricultural Sciences. LD05 was developed from the recipient inbred line Zheng58 by introducing the m2cryAb-vip3A fusion gene. This fusion gene was constructed by combining sequence- and structure-optimized fragments of cry1Ab and vip3Aa, broadening the insecticidal spectrum and enhancing target pest resistance [40].

**Figure 7:**
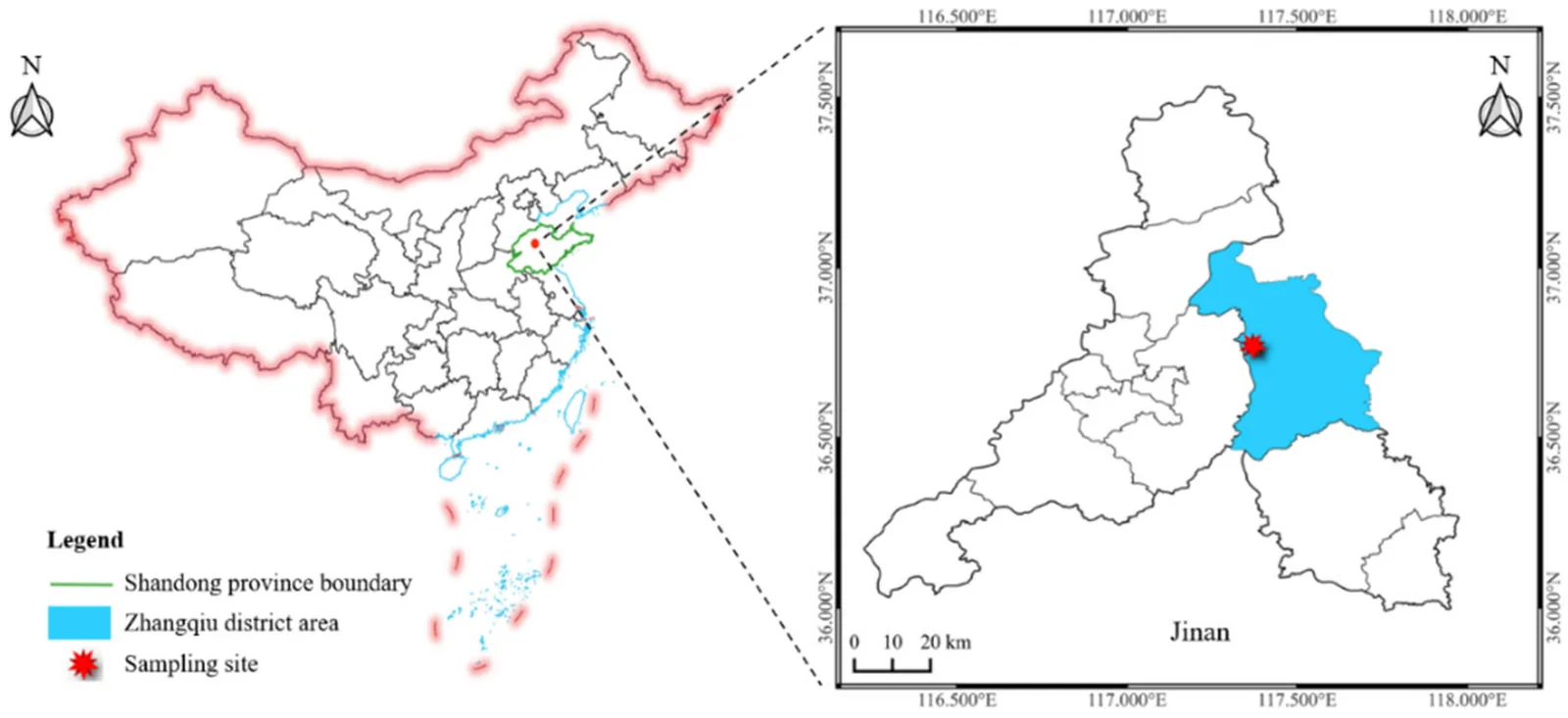
Location of the experiment sites in Zhangqiu District, Jinan City, Shandong Province, China. Sampling was conducted in the Huang-Huai-Hai Transgenic Maize Pilot and Industrialization Base during 2023 and 2024.

The experimental site was located in Dangjia Village, Longshan Subdistrict, Zhangqiu District, Jinan City, which belongs to the National Huang-Huai-Hai Transgenic Maize Pilot and Industrialization Base of the Ministry of Agriculture and Rural Affairs (Figure 7). Three treatments were established: Treatment 1, LD05 without application of the target herbicide; Treatment 2, LD05 sprayed with glufosinate (LD05(GA)); and Treatment 3, Zheng58 without herbicide application.

A randomized complete block design with three replicates was used [37,52]. Each plot had an area of 150 m^2^ with 1-m isolation strips (Figure S2). The same field site was used in 2023 and 2024 with treatments re-randomized within blocks annually. Maize was sown on 2 June 2023 and 11 June 2024. Maize was dibbled at 2–3 seeds per hole with 25 cm within-row and 60 cm between-row spacing. Seedlings were thinned to one plant per hole at the two-leaf-one-heart stage. No insecticides were sprayed throughout the entire growth period. Irrigation and fertilization, along with other agronomic measures, followed conventional local field management practices.

To avoid edge effects and minimize cross-contamination, all sampling was conducted in the inner area of each plot. A 1-m isolation strip may not fully prevent the movement of highly mobile arthropods between adjacent plots. However, the randomized complete block design, the use of independent plots, and the inner-plot sampling margins reduce the risk of cross-contamination. According to the Environmental Safety Detection of Genetically Modified Plants and Their Products—Insect-Resistant Maize—Part 4: Impact on Biodiversity (Ministry of Agriculture Announcement No. 953-10.4-2007) and the Environmental Safety Detection of Genetically Modified Plants and Their Products—Herbicide-Tolerant Maize—Part 4: Impact on Biodiversity (Ministry of Agriculture Announcement No. 953-11.4-2007), the population dynamics and community structure of non-target arthropods, the population dynamics of lepidopteran pests, and the occurrence of diseases in maize fields were assessed.

### 4.2. Investigation of Arthropods

Sampling was conducted at five points along the diagonal of each test plot. The independent experimental unit is the randomized plot. The five sampling points within each plot, the plants sampled at each point, and the repeated sampling occasions are subsamples or repeated measures, not independent replicates. For all statistical analyses, data were first averaged to the plot level before testing. Two survey methods were employed. For direct observation, 10 maize plants were investigated at each sampling point, and surveys were conducted every 7 days for a total of 10 times per plot. The species of all arthropods on the whole plant and on the ground surface were documented; at the start of each survey, the number of actively moving insects was quickly recorded, and specimens that could not be identified in the field were coded and brought back to the laboratory for further identification. For suction sampling, surveys were carried out every 10 days for a total of 5 times per plot. At each point, a suction sampler was used to collect all arthropods from five maize plants and from the ground surface within a 1-m^2^ area. The collected samples were returned to the laboratory, cleaned, preliminarily sorted, and preserved in 75% ethanol for further identification. For herbicide-treated plots, surveys were performed twice, at 2 and 4 weeks after application.

The following diversity indices were calculated: the Shannon–Wiener diversity index (*H*), the Pielou index (*J*), the Simpson dominance (*C*), the species richness (*S*) and the Community similarity index (*C′*).

The *H* of the arthropods was calculated using the following formula [39,53].

(1) $$H=-\sum_{i=1}^{S}P_{i}\ln P_{i}$$

The *J* of the arthropods was calculated using the following formula [39,53].

(2) J=H/lnS

The *C* of the arthropods was calculated using the following formula [39,53].

(3) $$C=\sum_{i=1}^{n}{\left(N_{i}/N\right)}^{2}$$

The *C′* of the arthropods was calculated using the following formula [39,53].

(4) $$C^{\prime }=2w/\left(a+b\right)$$

Species richness (*S*) was defined as the total number of arthropod species recorded within a defined region. *S* is the total number of species in a community. When S ≤ 1, *J* was undefined and excluded. *P_i_* is the proportion of individuals belonging to a particular species to the total number of individuals. *N_i_* is the number of individuals belonging to a particular species. *N* is the total number of individuals recorded in the study region. *w* is the number of species shared by two samples. a and b are the total numbers of species in each of the two samples, respectively.

### 4.3. The Expression Levels of M2CryAb-VIP3A and PAT

To detect the accumulation levels of the target proteins M2CryAb-VIP3A and PAT in LD05, tissue samples were collected at the whorl stage (V6–V7) and the mature stage (R5–R6). At the whorl stage, the third fully expanded leaf was sampled for the detection of both M2CryAb-VIP3A and PAT proteins; at the mature stage, kernels and stem segments from the first internode below the primary ear were collected exclusively for M2CryAb-VIP3A protein analysis. All samples were randomly collected from distinct randomized plots. All samples were flash-frozen in liquid nitrogen and stored at −80 °C for later use. Five individual plants sampled from the same plot within a given year were pooled as a single biological replicate, yielding a total of three replicates (each from a distinct plot). Each replicate was weighed, pooled, and subjected to protein extraction. The contents of M2CryAb-VIP3A and PAT proteins in transgenic maize LD05 were determined by double-antibody sandwich ELISA.

The M2CryAb-VIP3A ELISA kit was developed by Shanghai Youlong Biotechnology Co., Ltd. (Shanghai, China, AA0341), and the PAT ELISA kit was a commercial product Shanghai Youlong Biotechnology Co., Ltd. (AA1041). The M2CryAb-VIP3A protein standard was serially diluted with the sample extraction buffer to generate a calibration curve at concentrations of 5, 2.5, 1.25, 0.625, 0.3125, and 0.15625 ng/mL. Similarly, the PAT protein standard was serially diluted to concentrations of 3.2, 1.6, 0.8, 0.4, and 0.2 ng/mL. Specifically, for the M2CryAb-VIP3A assay, the LOD and LOQ were 0.06 ng/mL and 0.2 ng/mL, respectively. For the PAT assay, the LOD and LOQ were 0.6 ng/mL and 2 ng/mL, respectively. The samples were appropriately diluted with the extraction buffer to fall within the standard curve range. Leaf tissue (0.05 g) was extracted with 0.5 mL of buffer, while kernels and stem tissues (0.1 g each) were extracted with 1.0 mL of buffer. The mixtures were vortexed for 5 min, centrifuged at 4000 rpm for 3 min, and 100 μL of the supernatant was used for ELISA. Separate tools were used to prevent cross-contamination. All kits were equilibrated at room temperature for 30 min, and all reagents were shaken well before use. Technical wells were not counted as biological replicates.

The assay procedure was performed as follows: 100 μL of standards, blank controls (sample diluent), or samples were added to the corresponding wells, and the plates were sealed, gently mixed, and incubated at 37 °C in the dark for 60 min. The plates were then washed three times with 300 μL/well of wash buffer (10 s intervals). Subsequently, 100 μL of enzyme-labeled antibody working solution was added to each well, followed by another 60 min incubation at 37 °C in the dark. After washing five times (300 μL/well), 100 μL of chromogenic substrate was added, and the plates were incubated at 37 °C in the dark for 15 min. Finally, 50 μL of stop solution was added to each well, and the optical density (OD) was measured at 450 nm using a microplate reader (Thermo Fisher Scientific, Waltham, MA, USA) within 5 min. The measured values were calculated using interpolation based on the standard curves generated from the target protein standards. Specifically, standard curves were constructed using the mean values of the standards to obtain the regression equations (Figure S1). The concentrations of M2CryAb-VIP3A and PAT proteins in each sample were then determined by interpolation. Finally, the target protein contents were calculated based on the extraction volume and the sample dilution factor, and the results are expressed as μg target protein per gram of fresh weight (μg/g FW) [42].

### 4.4. Field Survey of LD05 Resistance to Major Lepidopteran Pests

Field surveys for target lepidopteran pests were conducted at the early whorl stage (V6–V8), late whorl stage (V10–VT), and maturity stage (R5–R6). In each plot, a diagonal five-point sampling method was employed, and at each sampling point, 20 maize plants from four adjacent rows (totaling 100 plants per plot) were continuously investigated, with sampling points spaced at least 5 m apart and avoiding border row plants. Each plot was surveyed independently, and all three replicate plots were investigated. The survey focused on the major local lepidopteran pests, including *Ostrinia furnacalis*, *Helicoverpa armigera* (Hübner), *Mythimna separata* (Walker), *Spodoptera exigua* (Hübner), *Proceras venosatus* (Walker), *Dichocrocis punctiferalis* (Guenée), and *Spodoptera frugiperda*. Lepidopteran pest damage was identified by typical symptoms across growth stages. At the early whorl stage, damage was assessed by marginal notches caused by *M. separata* and holes created by *H. armigera* and *S. exigua*. At the late whorl stage, *O. furnacalis* damage was identified by characteristic rows of perforations and frass on newly unrolled leaves. At the maturity stage, plants were dissected (including ears) to evaluate overall damage, characterized by external entry holes, internal feeding tunnels, and live larvae in stalks, as well as chewed kernels, severed silks, and frass in ears, with the number of entry holes and surviving larvae specifically recorded for *O. furnacalis*, *P. venosatus*, and *D. punctiferalis*. The grading criteria for lepidopteran pest damage were based on Ministry of Agriculture Announcement No. 953-10.4-2007 and No. 953-11.4-2007. Tunnel length was measured as the total tunnel length per 100 plants. All damage assessments were based on naturally infested plants; no artificial infestation was performed.

The control efficacy of LD05 against major lepidopteran pests was estimated as follows:

(5) E=(D−T)/D×100

*E* is the control efficacy (%), *D* is the number of damaged plants in the non-transgenic control plots, and *T* is the number of damaged plants in the LD05 plots.

### 4.5. Field Survey of Major Disease Incidence

Surveys were conducted once at the late whorl stage (V10–VT) and once at the maturity stage (R5–R6). In each plot, a diagonal five-point sampling method was adopted, and at each sampling point, 20 maize plants from two adjacent rows (10 plants per row) were continuously investigated, with sampling points placed at least 2 m from the field edge. The diseases surveyed included corn smut (*Ustilago maydis*), leaf spot (including northern and southern leaf blight), and southern rust (*Puccinia polysora*). Leaf spot included northern leaf blight and southern leaf blight. Diagnostic criteria were based on typical symptoms: northern leaf blight shows long, elliptical, grayish-green to brown lesions; southern leaf blight shows small, oval, reddish-brown lesions with a yellow halo. Disease severity for leaf spot and southern rust was assessed using a 0–9 scale: 0, no lesions; 1, <5% leaf area affected; 3, 5–10%; 5, 10–25%; 7, 25–50%; 9, >50% [54,55]. Corn smut was only assessed as incidence (%) [56].

The incidence of corn smut was expressed as disease incidence (*D*) and calculated using the following formula:

(6) D=N/T×100

*D* is the disease incidence (%). *N* is the number of diseased plants, and *T* is the total number of plants investigated.

The severity of leaf spot and southern rust was expressed as the disease index (*I*), calculated using the following formula [57]:

(7) $$I=\frac{\sum \left(N\times R\right)}{M\times T}\times 100$$

*I* is the disease index (%). *N* is the number of plants at a certain disease level. *R* is the disease level value. *M* is the maximum disease level value (*M* = 9), and *T* is the total number of plants investigated.

### 4.6. Weed Community Survey and Herbicide Efficacy Evaluation

In each plot, a diagonal five-point sampling method was adopted, with one 1-m^2^ quadrat established at each sampling point (five quadrats per plot), and sampling points were placed at least 2 m from the field edge. The herbicide was the Basta brand of glufosinate-ammonium, an 18% soluble concentrate (SL) and a non-selective contact foliar herbicide, applied at 3.75 L/ha. Glufosinate was applied at the V6–V7 stage about 20 days after sowing and sprayed in the late afternoon on a calm, rain-free day. Surveys were conducted four times: before glufosinate application (baseline survey), and at 14 days, 21 days after application, and at the tasseling stage (VT), with each replicate plot surveyed independently. At each survey, weed species, abundance, and coverage were recorded. Weed coverage was estimated visually as the percentage of the quadrat area covered by weed canopy. The pre-application survey served as baseline data to confirm consistency of weed occurrence across treatments. At each sampling date, comparisons of weed species composition, abundance, and coverage were made among three treatments—LD05 with glufosinate application (LD05 (GA)), LD05 without herbicide, and Zheng58 without herbicide—to determine whether the transgenic trait itself had any impact on weed occurrence. For all statistical analyses, data were first summarized and analyzed at plot level, so that the independent unit was the randomized plot.

The control efficacy of glufosinate against weeds was calculated using the following formula [58]:

(8) $$X=\frac{CK-PT}{CK}\times 100$$

*X* is the control efficacy (%). *PT* is the weed number in the treated plots (LD05 (GA)), and *CK* is the weed number in the control plots (LD05).

### 4.7. Statistical Analyses

All data are presented as mean ± standard deviation (SD), unless otherwise indicated. The independent experimental unit is the randomized plot. The five sampling points per plot, the plants sampled at each point, and the repeated survey dates are subsamples or repeated measures, not independent replicates. For further analysis, subsample data were averaged within each plot. To evaluate whether there are differences in the effects on biodiversity between transgenic maize LD05 and its non-transgenic control Zheng58 under identical environmental conditions (same developmental stage and field), one-way ANOVA was used to compare the differences among treatments. This was followed by Duncan’s multiple range test (DMRT) for post hoc multiple comparisons, in accordance with the guidelines of the Ministry of Agriculture of the People’s Republic of China Announcements No. 953-10.4-2007 and No. 953-11.4-2007. A value of *p* < 0.05 was considered a statistically significant difference, whereas *p* ≥ 0.05 indicated no significant difference. Two contrasts were explicitly defined: the genotype contrast (unsprayed LD05 vs. unsprayed Zheng58) and the herbicide contrast (sprayed LD05 vs. unsprayed LD05), with the latter compared only at matched dates after glufosinate application. Student’s t test was used to compare LD05 and Zheng58 for incidence of lepidopteran pests, disease incidence and disease index. In addition, to comprehensively evaluate differences in integrated community structure (arthropods, weeds, and diseases) between LD05 and Zheng58 fields, non-metric multidimensional scaling (NMDS) based on Bray–Curtis dissimilarity was performed using community characteristics of arthropods, weeds, and disease incidence as combined traits. PERMANOVA was used to test the significance of community structure differences between treatments [53,59]. By setting strata = year, the permutations were completely confined within each year. NMDS were performed using the Vegan package (v.2.6-4) in R software (v.4.4.3), and the results were visualized with ggplot2.

## Data Availability

The data that support the findings of this article are contained within the article and its Supporting Information published online.

## Supplementary Materials

The following supporting information can be downloaded at: https://www.mdpi.com/article/10.3390/plants15213329/s1, Figure S1: Representative standard curves for the ELISA detection of M2CryAb-VIP3A and PAT proteins; Figure S2: Field experimental layout in 2023 and 2024; Table S1: Statistics of 54 arthropod species; Table S2: Protein expression of M2CryAb-VIP3A (μg g^−1^ fresh weight); Table S3: Protein expression of PAT (μg g^−1^ fresh weight); Table S4: Insect damage plants (%); Table S5: Larvaes per 100 plants; Table S6: Tunnel per 100 plants (cm); Table S7: Disease index of southern corn rust (%); Table S8: Incidence of corn smut (%); Table S9: Disease index of corn leaf blight (%); Table S10: Weed control rate (%); Table S11: Weed species; Table S12: Number of weeds (weeds/m^2^); Table S13: Weed coverage (%); Code S1: Non-metric multidimensional scaling (NMDS) analysis of integrated community structure (arthropods, weeds, and diseases) in LD05 and Zheng58 fields.
