# Supplementary material for: Effect of Transgenic LD05 Maize Harboring the m2cryAb-vip3A Fusion Gene on Biodiversity in the Field
Source: Plants (Basel). 2026 Oct 28;15(21):3329. doi: 10.3390/plants15213329 (PMC13765917; doi:10.3390/plants15213329)
Supplement: Supplementary file 1 [file plants-15-03329-s001.zip › Supplementary materials.pdf]

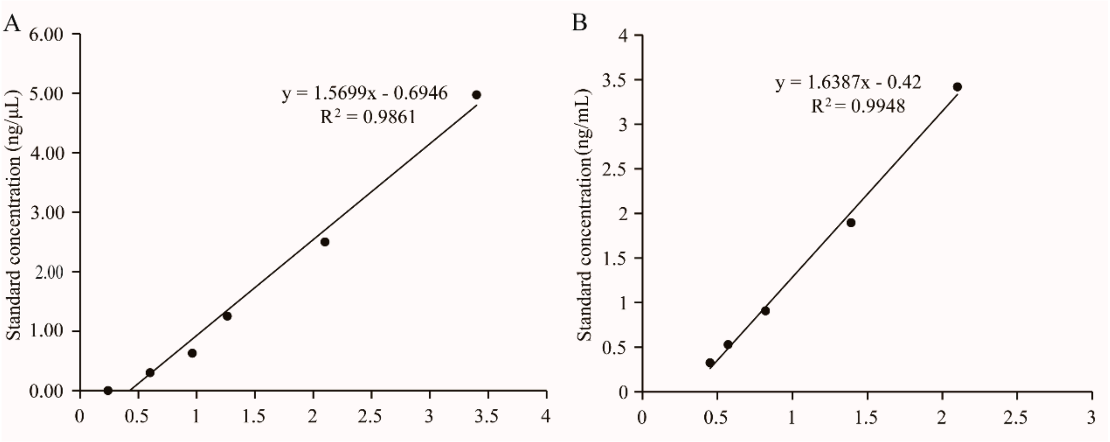

Supplementary Figure S1 Representative standard curves for the ELISA detection of M2CryAb-VIP3A and PAT proteins. (A) M2CryAb-VIP3A; (B) PAT.

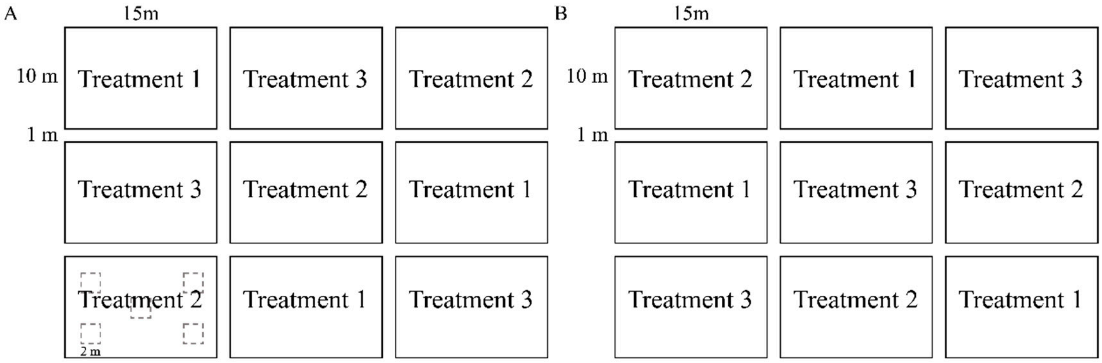

Supplementary Figure S2 Field experimental layout in 2023 and 2024. (A) 2023; (B) 2024. The dashed boxes in each plot represent sampling points, which are located 2 m from the plot edge.
